# Usefulness of a topical combination of dinotefuran and pyriproxyfen for long-term control of clinical signs of allergic dermatitis in privately-owned cats in Ile-de-France region

**DOI:** 10.1186/s13071-017-2335-x

**Published:** 2017-08-23

**Authors:** Odile Crosaz, Silvia Bonati, Amaury Briand, Elodie Chapelle, Noëlle Cochet-Faivre, Diane Ka, Céline Darmon-Hadjaje, Marie Varloud, Jacques Guillot

**Affiliations:** 1 Department of Parasitology, Mycology and Dermatology, CHUVA, École nationale vétérinaire d’Alfort, UPE, 7 avenue du Général de Gaulle, 94704, Maisons-Alfort, France; 2 Ceva Santé Animale, 10 avenue de la Ballastière, 33500, Libourne, France

**Keywords:** Dinotefuran, Pyriproxyfen, *Ctenocephalides felis*, Allergic dermatitis, Long-term control

## Abstract

**Background:**

The present study assessed the activity of a combination of dinotefuran and pyriproxyfen (Vectra® Felis) for long-term control (3 months) of allergic dermatitis (AD) in privately-owned cats under common household conditions in Ile-de-France region.

**Methods:**

This was an open pre-treatment *vs* post-treatment study. Twenty-eight client-owned cats with clinical signs of AD were enrolled in the study. They received topical application of the combination of dinotefuran and pyriproxyfen on days 0, 28, 56 and 84. Two parameters (clinical signs and pruritus severity) were used to assess the animals’ condition on days 0, 28 and 84. Fleas were counted if they were observed.

**Results:**

Of the 28 cats initially enrolled, 26 were presented on day 28 and 20 for the final evaluation on day 84. A significant improvement in clinical signs and pruritus was observed in cats for which fleas and/or flea feces were detected on day 0. Globally, the post-treatment AD clinical scores on days 28 and 84 were different from that of the pre-treatment on day 0, with a reduction of 30% and 71%, respectively. For cats with fleas and/or flea feces, the reduction on days 28 and 84 was 33% and 85%, respectively. The improvement of clinical signs and pruritus was not significant in cats with no visible fleas and no flea feces at the beginning of the trial (*n* = 8).

**Conclusions:**

The present study indicated that the treatment with a combination of dinotefuran and pyriproxyfen should be considered as useful in controlling fleas on cats without additional environmental treatment and useful for long-term control of clinical signs and pruritus in allergic cats.

**Electronic supplementary material:**

The online version of this article (doi:10.1186/s13071-017-2335-x) contains supplementary material, which is available to authorized users.

## Background

Allergic dermatitis (AD) is prevalent in cats, which includes the most frequent flea allergy dermatitis (FAD) and to a lesser extent hypersensitivity reaction towards food and environmental allergens [1]. FAD occurs in cats with repeated or continual exposure to saliva allergens of the flea species *Ctenocephalides felis*. Flea bites are responsible for irritation and pruritus [2, 3]. In cats, the clinical presentation of FAD is not pathognomonic but usually includes a pruritic and papular dermatitis. Because pruritus can be intense, excoriations and alopecia frequently develop. Generalized distribution is sometimes reported. Lesions of eosinophilic granuloma complex, including indolent ulcer, and symmetrical self-induced alopecia can also be found [2, 3].

To be effective in controlling flea infestation and signs of FAD, a parasiticide must combine high activity against adult fleas with a rapid onset of activity to minimize the number of flea bites and sufficient duration of action to prevent re-infestation [2, 3]. In addition, the ideal drug should be effective against immature flea stages in the environment [4, 5]. Dinotefuran belongs to the neonicotinoid family. It binds to nicotinic acetylcholine receptors resulting in depolarization of neurons and subsequent insect paralysis [6]. Dinotefuran kills fleas by contact. Pyriproxyfen is absorbed through the insect cuticle and acts as a juvenile hormones analogue [7]. The combination of dinotefuran and pyriproxyfen is available as a spot-application product (Vectra® Felis) for flea control in cats.

Considering that exposure to flea bites is the most frequent cause of AD in cats [1], we decided to evaluate the effect of a parasiticide, whose activity against fleas has been clearly demonstrated, on a population of cats with clinical signs suggestive of AD. We report here an open field study which assessed the effect of the treatment with a combination of dinotefuran and pyriproxyfen for long-term control (up to 3 months) of clinical signs of AD in privately-owned cats under common household conditions in Ile-de-France region.

## Methods

The study was an open pre-treatment *vs* post-treatment clinical field study. Cats were presented at the dermatology consultations of the Small Animal Hospital of Alfort Veterinary College (CHUVA, France) from April 2015 to July 2016.

Privately-owned cats with a clinical diagnosis of AD were recruited after a written informed consent was obtained from their owners. All cats came from Ile-de-France region and were each enrolled independently. During the study, cats were kept at home by their owners and fed and exercised according to their usual routine.

Cats were diagnosed with AD on the only basis of clinical signs [1]. All of them exhibited both pruritus and clinical problems, and/or signs including miliary dermatitis, eosinophilic granuloma complex, alopecia, erythema, papules and crusts. For most of the cats, a clinical history of chronic (more than one year) pruritus was reported.

Exclusion criteria were the following: (i) the cats were less than 7 weeks old or weighed less than 0.6 kg; (ii) an external antiparasitic treatment was administered within the month before presentation; (iii) the treatment of all animals in the household against fleas was not feasible; and (iv) cats had corticoids during the last month before inclusion.

During the first visit (day 0), the clinical history was collected and a standard clinical examination was made for each cat. The study protocol included additional visits on days 0, 28 (day 0 + 1 month) and 84 (day 0 + 3 months). At each visit, two parameters were used to evaluate the cat’s condition: (i) clinical signs of AD were globally and subjectively assessed as absent (0), mild (1), moderate (2) or severe (3) [8]; and (ii) pruritus was assessed using a scoring system, which includes intensity and frequency of pruritus and was originally developed for dogs [9] (Table 1). Fleas were counted if they were observed. For that purpose, each cat was combed for at least 5 min and combing continued until no further fleas had been found for 3 consecutive min.

**Table 1:** Pruritus grading scale, including frequency and intensity, with a 0–10 score [8]

Frequency of pruritus	Intensity of pruritus			
	Low	Moderate	Important	Severe
Occasional	1	2	3	4
Quite frequent	3	4	5	6
Frequent	5	6	7	8
Permanent	7	8	9	10

Each cat was treated with a topical application of the combination of dinotefuran and pyriproxyfen. The dose was based on the cat’s weight on day 0. All along the study, included cats received no concomitant treatment with any other anti-flea product or with any antipruritic or anti-inflammatory drug. Antibiotic or antiseptic could be used but only during the first month of the study. Other companion animals (cats and/or dogs) living at study households also received a monthly treatment either with a dinotefuran-pyriproxyfen spot on (Vectra® Felis) on cats, or dinotefuran-permethrin-pyriproxyfen spot on (Vectra® 3D) on dogs. Any observed health issues or adverse events following treatment were reported by the owners. The use of an antiparasitic spray or fogger to eliminate immature flea stages in the environment was decided by the veterinarian in case of heavy infestation.

At each time point, *t*, clinical signs (or pruritus severity) reduction was calculated using the arithmetic mean in the following formula: Clinical signs (or pruritus severity) reduction (%) = 100 × (mean day 0 – mean *t*)/mean day 0.

A mixed linear model, including day as a fixed effect, was selected to analyze clinical signs and pruritus severity values. For treatment comparisons, least squares means were calculated. The null hypothesis was that there was no significant difference in the testing parameters between the pre-treatment and post-treatment. Two tailed tests were used for the comparison. Statistical significance was declared when *P* ≤ 0.05. The primary software was IBM SPSS Statistics version 23 (IBM Co., Armonk, NY, USA).

## Results

Twenty-eight client-owned cats with AD were included in the study. They were mixed and pure-bred, ranging between 1 and 16 years old, and weighing between 2 and 7 kg. The enrolled population included three entire females, 14 spayed females, one entire male and 10 neutered males. There were 23 European cats, one exotic shorthair, one Persian, one Siamese, one Burmese and one Chartreux. Nineteen cats lived in a house with access to a garden (14 with frequent access to a garden, 5 with only limited access to a garden). Nine cats lived in an apartment. Eleven of the included cats were the only animals of the house, while 12 lived with another cat, two lived with a dog and three lived with cats and dogs. Clinical signs included miliary dermatitis (46% of the cats), head and neck pruritus (43%), symmetrical self-induced alopecia (43%) and eosinophilic granuloma complex (14%).

Of the 28 cats enrolled on day 0, 26 were presented on day 28, and 20 for the final clinical evaluation on day 84. Two cats were excluded from the study since they were not examined on both days 28 and 84. Six cats were presented on day 28 but not at the final clinical evaluation. The lack of availability of the cat-owners was the only reason for not presenting the animals.

On day 0, 8 cats had no fleas and no flea feces whereas 20 cats had fleas and/or flea feces. Among the 13 cats with fleas (1–40 fleas were detected per animal), the average flea number was 12.1. On day 28, 1–3 fleas were detected in four cats whereas on days 84, all the cats were flea-free.

Elimination of immature flea stages in the house (on day 0) was decided in only 5 of the cases. This number was too low to demonstrate any difference in response when comparing cats living in treated-houses and those living in non-treated houses.

At day 0, clinical signs of AD ranged between 1 and 3 with a mean of 2.3 (Table 2) and pruritus score ranged between 1 and 10 with a mean of 7.2 (Table 3).

**Table 2:** Evolution of the clinical scores in cats with or without fleas and/or flea feces (at the beginning of the trial)

Clinical score	All cats	Cats with fleas and/or flea feces at D0	Cats with no fleas and no flea feces at D0
Average			
Day 0	2.3 (*n* = 28)	2.4 (*n* = 20)	2.0 (*n* = 8)
Day 28	1.5 (*n* = 26)	1.6 (*n* = 19)	1.5 (*n* = 7)
Day 84	0.7 (*n* = 20)	0.4 (*n* = 14)	1.3 (*n* = 6)
Evolution			
Day 0 *vs* Day 28	mixed ANOVA, *df* = 69, *P* = 0.001	mixed ANOVA, *df* = 47, *P* < 0.0001	ns
Day 28 *vs* Day 84	mixed ANOVA, *df* = 69, *P* < 0.0001	mixed ANOVA, *df* = 47, *P* < 0.0001	ns
Day 0 *vs* Day 84	mixed ANOVA, *df* = 69, *P* < 0.0001	mixed ANOVA, *df* = 47, *P* < 0.0001	ns

*Abbreviations*: *df* degrees of freedom; *ns* no significant difference

**Table 3:** Evolution of the pruritus scores in cats with or without fleas and/or flea feces (at the beginning of the trial, D0)

Pruritus score	All cats	Cats with fleas and/or flea feces (*n* = 20 at D0)	Cats with no fleas and no flea feces (*n* = 8 at D0)
Average			
Day 0	7.2 (*n* = 28)	7.7 (*n* = 20)	6.0 (*n* = 8)
Day 28	4.3 (*n* = 26)	3.9 (*n* = 19)	5.3 (*n* = 7)
Day 84	2.8 (*n* = 20)	1.9 (*n* = 14)	5.0 (*n* = 6)
Evolution			
Day 0 *vs* Day 28	mixed ANOVA, *df* = 69, *P* < 0.0001	mixed ANOVA, *df* = 47, *P* < 0.0001	Ns
Day 28 *vs* Day 84	mixed ANOVA, *df* = 69, *P* = 0.044	mixed ANOVA, *df* = 47, *P* = 0.012	Ns
Day 0 *vs *Day 84	mixed ANOVA, *df* = 69, *P* < 0.0001	mixed ANOVA, *df* = 47, *P* < 0.0001	Ns

*Abbreviations*: *df* degrees of freedom, *ns* no significant difference

Over the study period, a significant improvement in clinical signs was observed in cats for which fleas and/or flea feces were detected on day 0 (Table 2) (Additional file 1: Fig. S1). Globally, the post-treatment AD clinical scores on days 28 and 84 were different from that of the pre-treatment on day 0 with a reduction of 30% and 71%, respectively. For cats with fleas and/or flea feces, the reduction on days 28 and 84 was 33% and 85%, respectively. A significant improvement in pruritus was observed in cats for which fleas and/or flea feces were detected on day 0 (Table 3). Globally, the post-treatment pruritus severity scores on day 28 and 84 were different from those of the pre-treatment day 0 with a reduction of 39% and 61%, respectively. For cats with fleas and/or flea feces, the reduction on days 28 and 84 was 46% and 75%, respectively.

No health issue was reported in any cat after any of the three applications of the combination of dinotefuran and pyriproxyfen at days 0 and 84.

## Discussion

The present study was about the activity of a topical combination of dinotefuran-pyriproxyfen to control clinical signs of AD in privately-owned cats in France. When the cats were enrolled, the diagnosis of FAD could not be established with certainty. However, the administrations of the antiparasitic drug were followed by a significant improvement of clinical signs and pruritus in cats with visible fleas and/or flea feces (*n* = 20), suggesting retrospectively that these animals had FAD. For cats with no fleas and no flea feces at the beginning of the study, post-treatment AD clinical and pruritus scores on days 28 and 84 were not significantly different from those of the pre-treatment on day 0.

Cats included in the present study were not skin-tested with flea allergens. Intradermal injection of flea antigens or serological tests was used to diagnose FAD in dogs [9–11] and cats [12]. However, positive immediate intradermal reactivity to flea antigens is possible in normal dogs. In the study of Kunkle et al. [11], a false-positive reaction was observed in 24% of dogs. Laffort et al. [9] suggested that skin tests with pure flea saliva provided the best correlation between the clinical approach to FAD diagnosis and intradermal reactivity. In cats, Bond et al. [13] compared the results of a serological test and intradermal reactivity with different commercially available *C. felis* antigens. A challenge with living fleas was used to assess the presence or absence of sensitization to *C. felis*. Considering this “gold standard”, Bond et al. [13] were able to calculate the sensitivity of the serological test (0.88) and that of the intradermal tests (0–0.33).

The scoring feline allergic dermatitis (SCORFAD) was proposed for the assessment of disease severity and evaluation of therapeutic response in trials on feline allergic dermatitis [14]. Feline dermatitis extent and severity index (FeDESI) is another scoring system for the evaluation of feline hypersensitivity [15]. In a comparative analysis, Noli & Cena [16] indicated that SCORFAD was more difficult to complete than FeDESI. In the present study, we decided to use a global and intuitive score which was similar to that adopted by Paarlberg et al. for naturally-flea infested cats [17] and by Dickin et al. for experimentally-flea infested cats [8].

It is always difficult to assess the severity of pruritus in companion animals. To date, only the Pruritus Visual Analog Scale (PVAS) has been validated in dogs [18]. This scale was used in a recent investigation about canine FAD in Ile-de-France region [19]. In the present study, we decided to use a pruritus grading scale including frequency and intensity [9]. However this scale has not been validated. Other studies may be necessary to evaluate this pruritus grading scale for cats. Recently, Noli & Cena [16] failed to demonstrate any correlation between FeDESI, SCORFAD and PVAS.

Several different insecticides (or combinations of insecticides) were shown to be useful for the control the clinical signs of AD, including FAD in cats, but there are only a few studies about the control of clinical signs in naturally infested cats [12, 17, 20–22]. For an efficient control of flea allergy, two major characteristics are required: (i) a rapid adulticide activity; and (ii) a long duration of action. The dinotefuran-pyriproxyfen spot on, used in the present study, meets both criteria: dinotefuran kills fleas as early as 1 h after application, with more than 95% reduction in flea numbers by 2 h [23], and approximately 91–98% reduction in flea numbers for a 30-day period. The results of the present study confirmed that the combination of dinotefuran and pyriproxyfen should be recommended for cats suffering from AD, including FAD. Both the clinical and the pruritus scores improved during the study. This was significant only in the population of cats with fleas and/or flea feces at inclusion. Enrolled cats, either with or without visible fleas or flea feces, may have concurrent atopy and/or food hypersensitivity, which can greatly complicate the diagnostic workup as well as the therapeutic regimen. As a conclusion, the improvement reported in the present study could have been due to flea control in flea-infested allergic, but not necessarily flea-allergic, cats. Furthermore, spontaneous improvement of included cats cannot be ruled out because the present investigation was an open, uncontrolled study.

## Conclusions

Monthly treatments with the combination of dinotefuran and pyriproxyfen should be considered as useful in controlling fleas on cats without additional environmental treatment and useful for long-term control of clinical signs and pruritus in cats with AD.

## Additional file

Additional file 1: Figure S1. A 9-year-old cat before (day 0) and after (day 84) monthly application of the combination of dinotefuran and pyriproxyfen. Fleas (*n* = 5) and flea feces were detected at day 0. (DOCX 1235 kb)

## Abbreviations

AD: Allergic dermatitis
CADESI: Canine atopic dermatitis extend severity index
FAD: Flea allergy dermatitis
FeDESI: Feline dermatitis extent and severity index
SCORFAD: Scoring feline allergic dermatitis
