# Supplementary material for: Usefulness of a topical combination of dinotefuran and pyriproxyfen for long-term control of clinical signs of allergic dermatitis in privately-owned cats in Ile-de-France region
Source: Parasit Vectors. 2017 Aug 23;10:392. doi: 10.1186/s13071-017-2335-x (PMC5567644; doi:10.1186/s13071-017-2335-x)
Supplement: Additional file 1: Figure S1. — A 9-year-old cat before (day 0) and after (day 84) monthly application of the combination of dinotefuran and pyriproxyfen. Fleas (n = 5) and flea feces were detected at day 0. (DOCX 1235 kb) [file 13071_2017_2335_MOESM1_ESM.docx]

**Figure S1** =

A 9-year-old cat before (day 0) and after (day 84) monthly application of the combination of dinotefuran and pyriproxyfen. Fleas (n=5) and flea feces were detected at day 0.

| **Day 0** | **Day 84** |
| --- | --- |
| 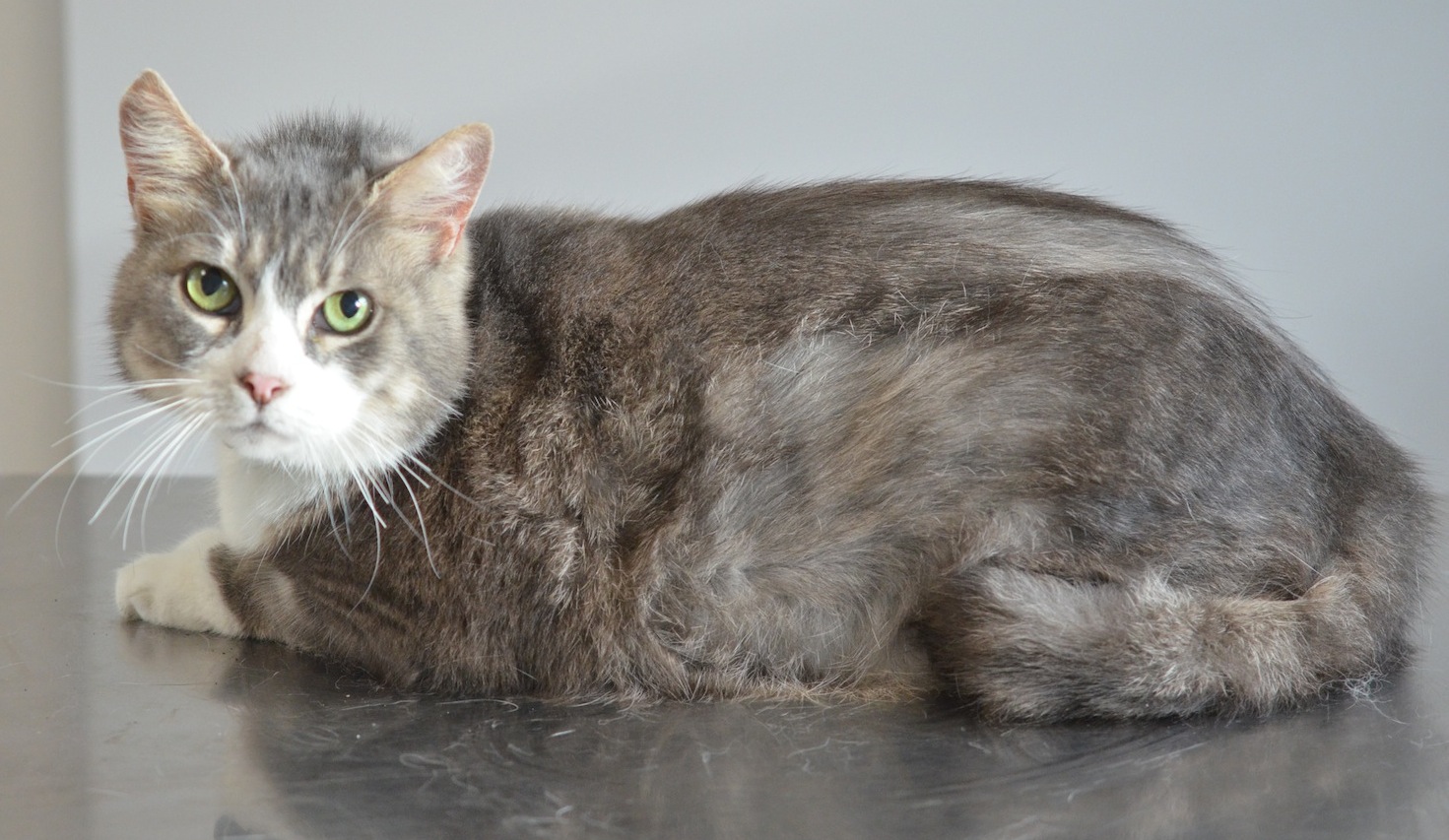 | 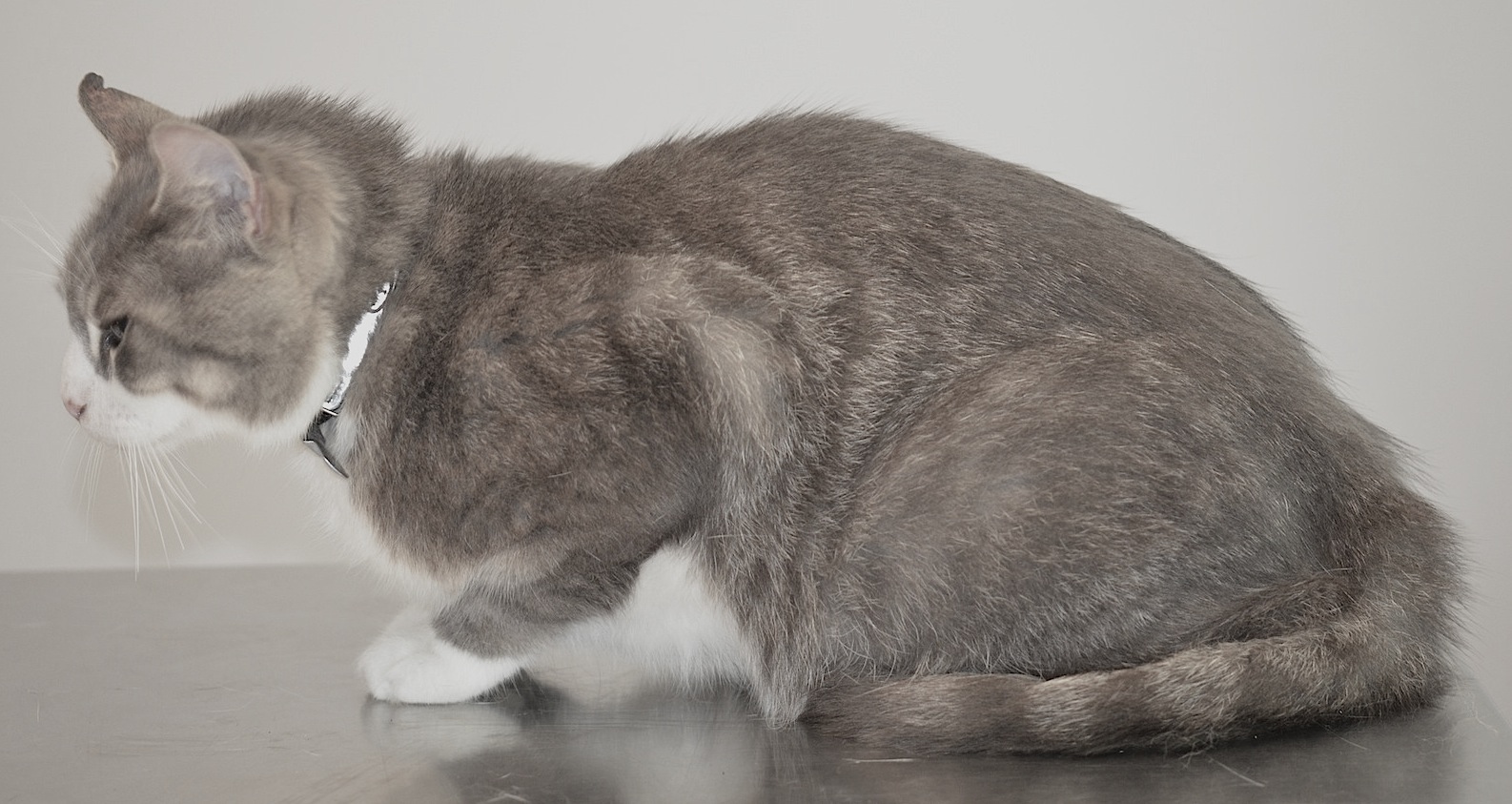 |
| 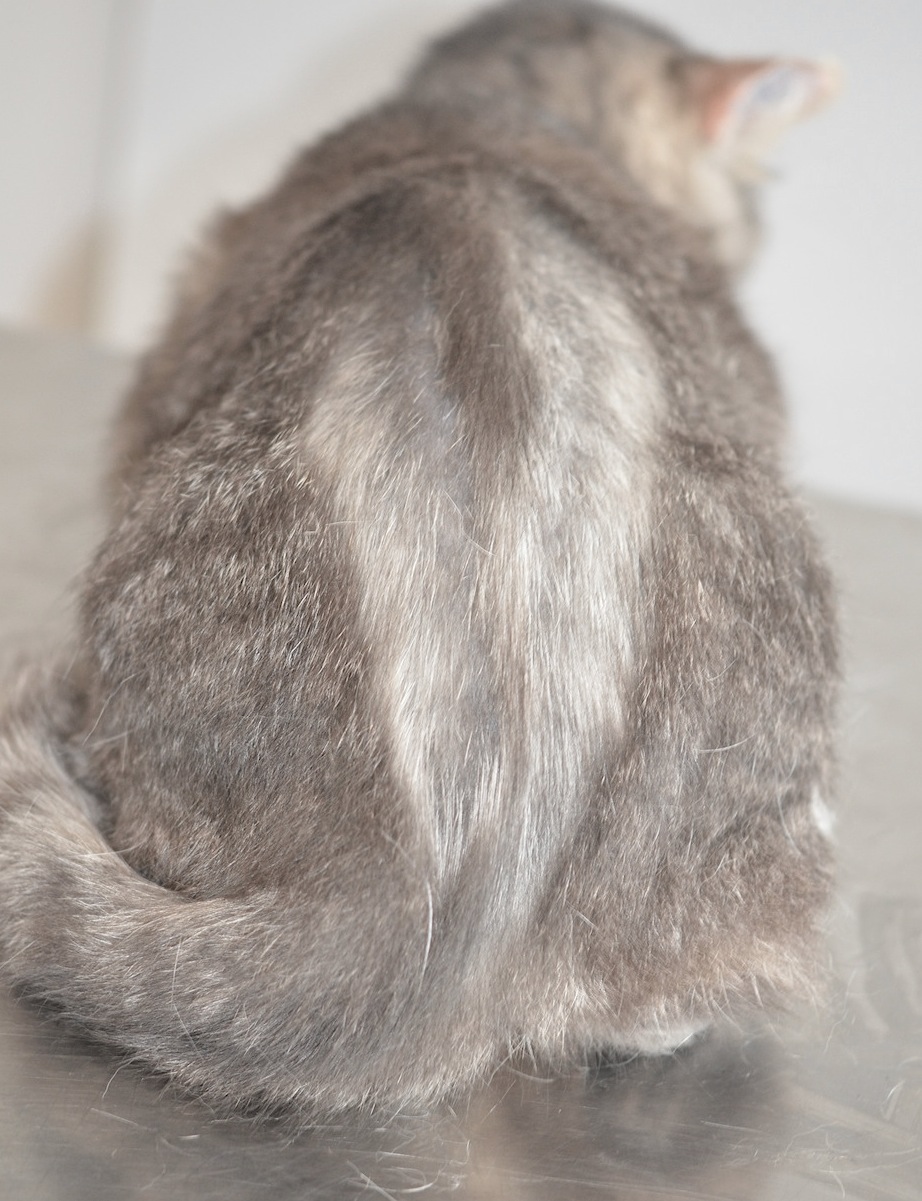 | 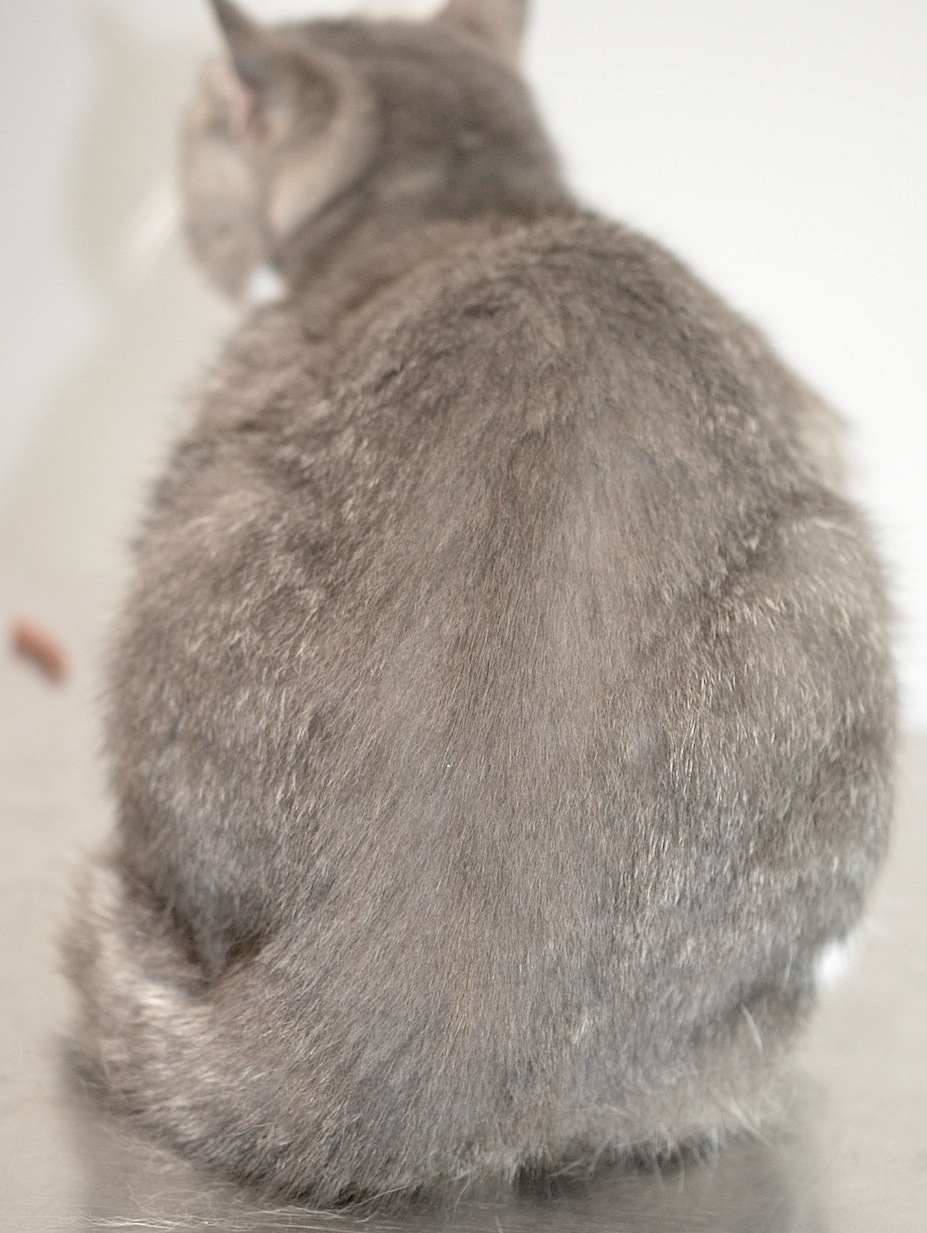 |
